# Actin Family Proteins in the Human INO80 Chromatin Remodeling Complex Exhibit Functional Roles in the Induction of Heme Oxygenase-1 with Hemin

**DOI:** 10.3389/fgene.2017.00017

**Published:** 2017-02-21

**Authors:** Yuichiro Takahashi, Hirokazu Murakami, Yusuke Akiyama, Yasutake Katoh, Yukako Oma, Hitoshi Nishijima, Kei-ichi Shibahara, Kazuhiko Igarashi, Masahiko Harata

**Affiliations:** ^1^Laboratory of Molecular Biology, Graduate School of Agricultural Science, Tohoku UniversitySendai, Japan; ^2^Department of Biochemistry, Graduate School of Medicine, Tohoku UniversitySendai, Japan; ^3^Department of Integrated Genetics, National Institute of GeneticsMishima, Japan; ^4^Division of Molecular Immunology, Institute of Advanced Medical Sciences, Tokushima UniversityTokushima, Japan; ^5^Japan Agency for Medical Research and DevelopmentTokyo, Japan

**Keywords:** actin family, chromatin, chromatin remodeling, heme oxygenase-1, stress response

## Abstract

Nuclear actin family proteins, comprising of actin and actin-related proteins (Arps), are essential functional components of the multiple chromatin remodeling complexes. The INO80 chromatin remodeling complex, which is evolutionarily conserved and has roles in transcription, DNA replication and repair, consists of actin and actin-related proteins Arp4, Arp5, and Arp8. We generated Arp5 knockout (KO) and Arp8 KO cells from the human Nalm-6 pre-B cell line and used these KO cells to examine the roles of Arp5 and Arp8 in the transcriptional regulation mediated by the INO80 complex. In both of Arp5 KO and Arp8 KO cells, the oxidative stress-induced expression of *HMOX1* gene, encoding for heme oxygenase-1 (HO-1), was significantly impaired. Consistent with these observations, chromatin immunoprecipitation (ChIP) assay revealed that oxidative stress caused an increase in the binding of the INO80 complex to the regulatory sites of *HMOX1* in wild-type cells. The binding of INO80 complex to chromatin was reduced in Arp8 KO cells compared to that in the wild-type cells. On the other hand, the binding of INO80 complex to chromatin in Arp5 KO cells was similar to that in the wild-type cells even under the oxidative stress condition. However, both remodeling of chromatin at the *HMOX1* regulatory sites and binding of a transcriptional activator to these sites were impaired in Arp5 KO cells, indicating that Arp5 is required for the activation of the INO80 complex. Collectively, these results suggested that these nuclear Arps play indispensable roles in the function of the INO80 chromatin remodeling complex.

## Introduction

In the nucleus of eukaryotes, the genomic DNA is packaged into a complex nucleoprotein structure, known as chromatin. Chromatin structure restricts the accessibility of factors involved in the DNA-based cellular process, including transcription, DNA replication, and DNA damage repair. The ATP-dependent chromatin-remodeling complexes regulate these processes through remodeling of the chromatin structure. These complexes consist of an enzymatic component and multiple subcomponents, which regulate their complex functions. It is known that some of the chromatin remodeling complexes contain actin family proteins as essential subcomponents (Oma and Harata, 2011).

The actin family consists of conventional actin and actin-related proteins (Arps). Arps are classified into Arp1 to Arp10 subfamilies, and each subfamily is evolutionarily conserved (Dion et al., 2010; Oma and Harata, 2011). The structure of the core domain of actin and Arp molecules, known as the actin fold, is highly conserved among the members of the actin family (Fenn et al., 2011; Gerhold et al., 2012). On the other hand, each member of this family has distinct molecular surface structure. Whereas some of the Arps are predominantly localized in the cytoplasm, several Arps (namely Arp 4, Arp 5, Arp 6, Arp 7, Arp 8, and Arp 9) are accumulated in the nucleus (Dion et al., 2010; Oma and Harata, 2011). Consistent with these results, all of these nuclear Arps were found to be parts of chromatin remodeling complexes of which actin is also a component.

The INO80 chromatin remodeling complex contains four actin family members: actin, Arp4, Arp5, and Arp8 (Oma and Harata, 2011; Tosi et al., 2013). These actin family proteins of the INO80 complex is evolutionarily conserved from yeast to human (Cai et al., 2007; Wu et al., 2007; Yao et al., 2008). Although actin and Arp4 are also found in some other chromatin remodeling complexes, Arp5 and Arp8 are identified as specific components of the INO80 complex. In addition to containing the Ino80 molecule as a scaffold, the budding yeast INO80 complex also contains four topological modules, namely Arp8-, Arp5-, Nhp10-, and Rvb-modules (Tosi et al., 2013).

Arp8 is included in the Arp8-module together with actin and Arp4, and has histone binding ability (Shen et al., 2003; Gerhold et al., 2012). We have recently reported that the human Arp8 binds to double- and single-stranded DNAs (Osakabe et al., 2014). We have also established gene knockout (KO) human cell line lacking Arp8 (Arp8-KO cells), and showed that Arp8 is required for the double strand DNA break (DSB) repair function of the INO80 complex (Osakabe et al., 2014). However, human Arp8's contribution to transcriptional regulation remains yet to be analyzed using the Arp8KO cells. Although Arp5, which is included in the Arp5-module, a module different from the Arp8-module, is supposed to have a distinctive role in the INO80 complex (Kitamura et al., 2015), our knowledge regarding the function of the human Arp5 in the INO80 complex is still very limited, especially its role in transcriptional regulation.

Reactive oxygen species (ROS), which are highly reactive in nature and cause oxidative stress, are known to disturb cellular homeostasis, which in some cases lead to the development of cancer and other diseases (Loboda et al., 2016). ROS are known to induce transcription of a set of genes, and heme oxygenase-1 (HO-1), which is a representative oxidative stress-inducible protein, has potent anti-inflammatory, antioxidative and antiproliferative effects (Vile et al., 1994; Camhi et al., 1995; Maeshima et al., 1996). Recently, Katoh et al. (2011) observed that Ino80 and Arp4 in complexes associate with the promoter of *HMOX1* gene, which encodes for HO-1, suggesting a possibility that the INO80 complex is involved in the transcriptional regulation of *HMOX1*.

In this study, we examined the roles of human Arp5 and Arp8 in the transcriptional regulation mediated by the INO80 complex using respective KO cells. We observed that these actin family proteins have distinctive roles in the activation of *HMOX1*. Using ChIP analysis we found that Arp8, but not Arp5, is required for the binding of INO80 complex to chromatin. On the other hand, both remodeling of chromatin at the *HMOX1* regulatory sites and binding of a transcription activator to these regulatory sites were impaired in Arp5 KO cells. These observations provided novel information about the distinctive functional contributions of actin family proteins in the transcriptional regulation mediated by the human INO80 complex.

## Materials and methods

### Cell culture and induction of oxidative stress

Nalm-6 pre-B cells were cultured at 37°C in Roswell Park Memorial Institute medium containing GlutaMAX™-I (Invitrogen) supplemented with 10% fetal bovine serum, penicillin, and streptomycin as described previously (Ono et al., 2009). To induce oxidative stress, hemin (Sigma) was added to the culture medium to a final concentration of 20 μM.

### Establishment of Arp5-knockout (KO) cells

Arp5 KO cells were generated using protocols similar to that was used for the generation of Arp8 KO cells (Osakabe et al., 2014) with some modifications. Briefly, the left (3.1 kb) and right (3.6 kb) arms of the targeting vectors were, respectively, amplified by PCR using the genomic DNA purified from Nalm-6 pre-B cells as the template. The left arm and the right arm contained exons 2–3 and intron 5, respectively (see Supplementary Figure S1). The disruption of both alleles of the *ARP5* gene was confirmed by Southern blot analysis using probes shown in Supplementary Figure S1. Arp5 knockout cells were successfully established, and they were confirmed to be devoid of Arp5 by Western blot analysis using an anti-Arp5 antibody.

### Western blot analysis

Western blot analysis was performed as was described earlier (Kitayama et al., 2009) using anti-Arp5 (Kitayama et al., 2009), anti-Ino80 (Abcam), anti-α-tubulin (Sigma), anti-MafK (Santa Cruz Biotechnology), anti-Bach1 (Dohi et al., 2008), or anti-Nrf2 (Santa Cruz Biotechnology) antibody. An anti-IgG conjugated to horseradish peroxidase (Promega) was used as the secondary antibody, and ECL Western blotting detection reagents (GE Healthcare) were used for the detection of bound antibodies.

### Quantitative RT-PCR analysis

Total RNA from human Nalm-6 pre-B cells was extracted with the RNeasy Mini kit (QIAGEN) following the manufacture's protocol. To prepare cDNA, an aliquot (2.0 μg) of the extracted RNA was incubated with 10 μl of 2xMaster Mix (Applied Biosystems) (RT buffer, 100 mM dNTP Mix, 10 mM random primers, 1 μl Multiscribe Reverse Transcriptase, RNase inhibitor, nuclease-free water) for 10 min at 25°C, for 120 min at 37°C and finally for 5 min at 85°C. An aliquot of this cDNA was used for quantitative PCR as described (Kusakabe et al., 2016). Quantitative PCR was carried out using gene specific primers for human *HMOX1* (5′-CTCTCGAGCGTCCTCAGC-3′ and 5′-TTCAGGGCCTCTGACAAATC-3′) and for human *36B4* (5′-CGACCTGGAAGTCCAACTAC-3′ and 5′-ATCTGCTGCATCTGCTTG-3′).

### Chromatin immunoprecipitation (ChIP) assay

ChIP assays was performed as described earlier (Kimura et al., 2008) with some modifications. In brief, Nalm-6 pre-B cells (6–9 × 10^7^ cells in a 100 ml medium) were mixed with 2.7 ml formaldehyde (the final concentration of 1%) and shaken at room temperature for 5 min to crosslink proteins to DNA. To stop the crosslinking reaction, 17.5 ml of 2M glycine was added, and the cells were first washed with 30 ml PBS and then with 10 ml NP-40 buffer [10 mM Tris-HCl (pH 8.0), 10 mM NaCl and 0.5% NP-40]. The cells were resuspended in 200 μl SDS lysis buffer [50 mM Tris-HCl (pH 8.0), 10 mM EDTA, and 1% SDS], and to this 400 μl ChIP dilution buffer [50 mM Tris-HCl (pH 8.0), 167 mM NaCl, 1.1% Triton X-100 and 0.11% sodium deoxycholate] was added. This mixture was sonicated using a Bioruptor (CosmoBio) for the shearing of the chromatin (~500 bp). After removing insoluble materials by centrifugation (12,000 g, 5 min), 200 μl ChIP dilution buffer was added to the supernatant to generate the input fraction for the ChIP analysis. Protein A- and G-Dynabeads (Invitrogen; 15 μl suspension of each) were washed and mixed with the ChIP input fraction and a specific antibody to the protein of interest. This mixture was incubated overnight at 4°C with rotation. Following the incubation, beads were washed sequentially with 1 ml of RIPA [50 mM Tris-HCl (pH 8.0), 1 mM EDTA, 0.1% SDS, 1% Triton X-100, 0.1% sodium deoxycholate]-150 mM NaCl buffer, 1 ml RIPA-500 mM NaCl buffer, and 1 ml TE [10 mM Tris-HCl (pH 8.0) and 1 mM EDTA]. After removing TE, beads were directly mixed with 200 μl elution buffer [10 mM Tris-HCl (pH 8.0), 300 mM NaCl, 5 mM EDTA, 0.5% SDS] and incubated overnight at 65°C to reverse the cross-linking. Immunoprecipitated DNA was purified and analyzed by real-time PCR (ABI PRISM 7000 or CFX Connect Real-Time System). Obtained values were normalized with that of DNA prepared from the input fraction. Sequences of PCR primers used for the real-time PCR were: HO-1 E1 (5′-CAGTGCCTCCTCAGCTTCTC-3′ and 5′-CTCGGTGGATTGCAACATTA-3′), HO-1 E2 (5′-CTCTGCCCCTGCTGAGTAAT-3′ and 5′-GAGCAGCTGGAACTCTGAGG-3′), Rad54B intron9 (5′-TAGCTGGGACTGCAGGTGTA-3′ and 5′-GTATTGCCAGGCCACAAGAT-3′).

### Nuclease accessibility assay

Nuclease accessibility assay was performed using the EpiQ chromatin analysis kit (BIO-RAD). Permeabilized Nalm-6 pre-B cells were treated with the nuclease supplied in the kit, the DNA was purified after the nuclease treatment, and the purified DNA was amplified using the region specific primers and reagents supplied in the EpiQ chromatin analysis kit according to the protocol provided by the manufacturer of the kit. Sequences of PCR primers for the E1, E2, exon5 of *HMOX1* used in this assay were indicated above under ChIP Assay. Sequences of other primers used in this assay were: HO-1 promoter (5′-GGGGGCTCTGGAAGGAGCAAAATCA-3′ and 5′-CAGTGTGGGGTGGAGAGGAGCAGTC-3′), GAPDH promoter: (5′-CGCACGTAGCTCAGGCCTCAAGACC-3′ and 5′-GGCTGACTGTCGAACAGGAGGAGCA-3′).

## Results

### Establishment of an Arp5 gene knockout human cell line

We generated human Arp5 KO cells and utilized them to analyze the role of Arp5 in the INO80 chromatin remodeling complex. Both the alleles of the *ARP5* gene in the human Nalm-6 pre-B cell line were disrupted using conventional gene knockout methods and an Arp5 KO cell line was established (Supplementary Figure S1). Western blot analysis by using a specific anti-Arp5 antibody confirmed that no Arp5 was expressed in Arp5 KO cells (Supplementary Figure S2A). Knockout of *ARP5* gene did not bestow lethal phenotype in Arp5 KO cells; however, the growth of Arp5 KO cells was slightly slower than that of the wild-type cells (Supplementary Figure S2B). These Arp5 KO cells, along with Arp8 KO cells (Osakabe et al., 2014), were used for further analyzing the function of INO80 complex.

### INO80 complex is involved in oxidative stress-induced expression of *HMOX1*

**Figure 1:**
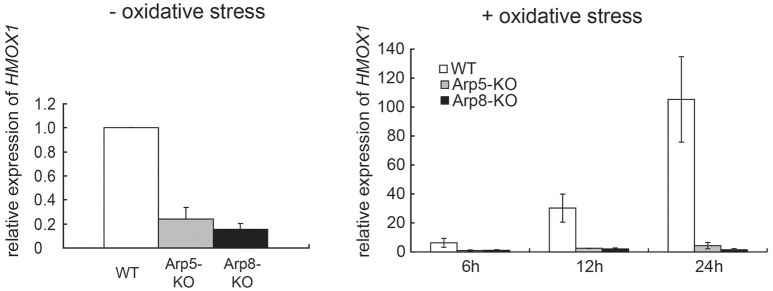
**The INO80 complex is involved in *HMOX1* expression**. Quantitative RT-PCR (qRT-PCR) analysis of *HMOX1* mRNA in wild-type (WT), Arp5 KO, and Arp8 KO cells. Left panel: control cells (not treated with hemin) and Right panel: cells treated with 20 μM hemin, an oxidative stress inducer, for the indicated time. The expression level of *HMOX1* in wild-type control cells (without oxidative stress) was arbitrarily set at 1. The expression of 36B4 gene was used to normalize the results. Averages of at least three independent experiments (± standard deviation) are shown.

To determine whether the INO80 complex is involved in transcriptional regulation of *HMOX1*, we analyzed the expression levels of *HMOX1* mRNA in wild-type, Arp5 KO, and Arp8 KO cells. The expression level of *HMOX1* was moderately decreased both in Arp5 KO and Arp8 KO cells in the absence of oxidative stress with hemin (Figure 1, left panel). However, significantly decreased expression levels of *HMOX1* were observed in both KO cells, as compared to that in the wild-type cells, following induction with oxidative stress; notably, the expression level of *HMOX1* in Arp5 KO and Arp8 KO cells was ~1/100 of that of the wild-type cells (Figure 1, right panel). This result suggested that the INO80 complex is required for the induction of *HMOX1* expression with hemin and that Arp5 and Arp8 may have essential roles in INO80complex function.

**Figure 2:**
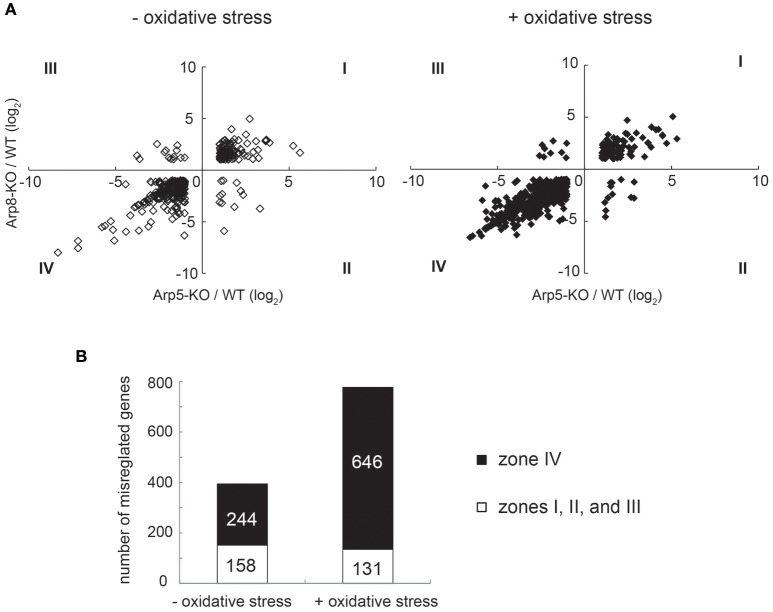
**Microarray analysis of Arp5 KO and Arp8 KO cells induced with or without oxidative stress**. **(A)** Genes whose transcriptional changes were significant in Arp5 KO and Arp8 KO cells under control (without oxidative stress, left panel) and experimental (with oxidative stress, right panel) conditions were plotted according to their log_2_ ratios. **(B)** Zone plot of transcript expression. Genes which were misregulated in these KO cells were counted and number of genes expressed in each zone, as shown in **(A)** above, were plotted. The filled bar indicates the number of genes in zone IV, expression levels of which were down-regulated both in Arp5 KO and Arp8 KO cells. The open bar indicates total number of other misregulated genes (Zones I, II, and III) in Arp5-KO and Arp8-KO cells.

We also performed a genome-wide microarray transcription analysis of control (without oxidative stress) and oxidative stress-induced Arp5-KO and Arp8-KO cells. The relative change in transcription of each gene in the gene loci was calculated by comparing its transcription level in Arp5 KO or Arp8 KO cells with that in the wild-type cells. The raw data and experimental details were deposited in the GEO database under accession number GSE66888. We plotted the degree of change in the transcription level of each gene whose transcription was misregulated in Arp5 KO or Arp8 KO cells (>2-fold) (Figure 2A). The genes belonging to the groups I to IV under each condition are listed in Supplementary Tables S1–S8. While analyzing the results obtained from the cells without oxidative stress (Figure 2A, left panel) and cells induced with oxidative stress (Figure 2A, right panel), we found a correlation between the degree of misregulation observed in Arp5 KO (horizontal axis) and Arp8 KO (vertical axis) cells (*R*^2^ = 0.64 and 0.77 in the absence and presence of oxidative stress, respectively); more than 90% of the misregulated genes were found either in zone I (upregulated in both cell types) or in zone IV (downregulated in both cell types) in the zone plot of transcript expression (Figure 2B). This observation suggested that both Arp5 and Arp8 are required for the transcriptional regulation mediated by the INO80 complex. Interestingly, the number of genes found in the zone IV of the zone plot were significantly increased following the induction with oxidative stress (Figure 2B, *p* = 1.9 × 10^−17^). In the absence of oxidative stress, genes belonging to eight GO categories are significantly accumulated (*p* < 0.01) in the zone IV (Supplementary Table S9). On the other hand, in the presence of oxidative stress, genes belonging to 48 GO categories are significantly accumulated in the zone IV (Supplementary Table S10). Genes for heme oxygenase 1 *(HMOX1*), glutathione peroxidase (*GPX3*), and glutathione S-transferase (*GSTM3*) are included in the zone IV only in the presence of oxidative stress. These observations suggest that the INO80 complex is involved in the expression of not only typical oxidative stress genes but also many other genes.

### Arp8, but not Arp5, is involved in the binding of Ino80 to chromatin

**Figure 3:**
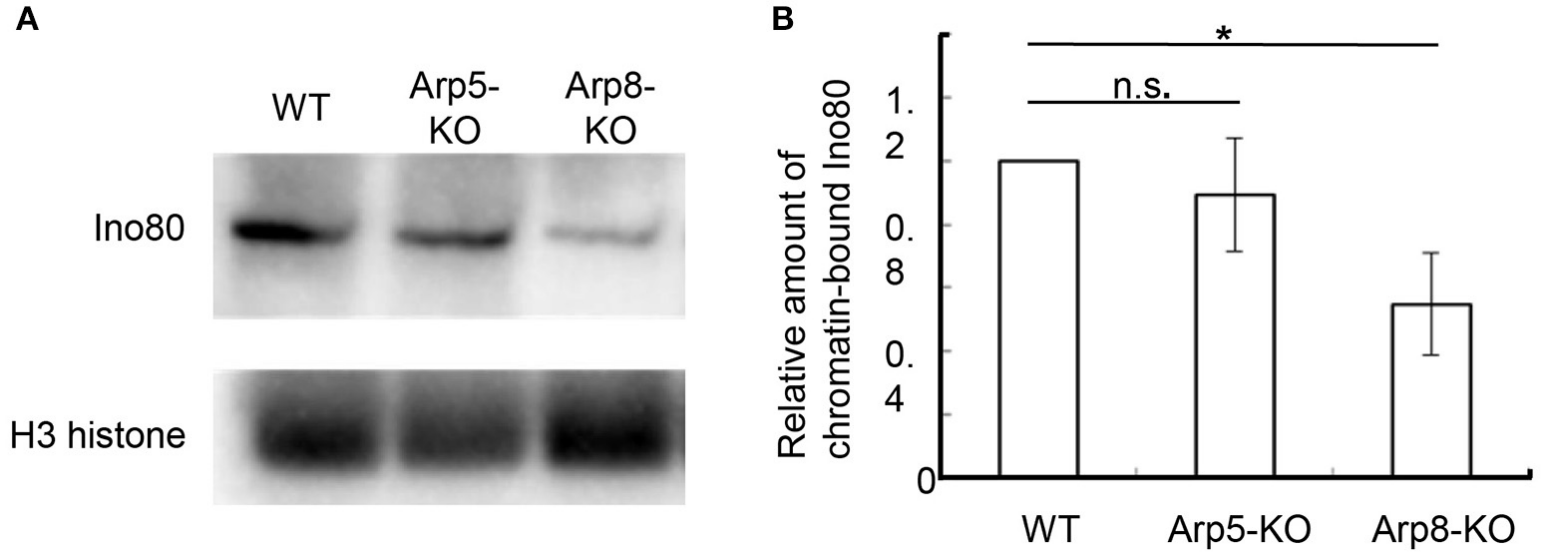
**Quantification of chromatin-bound Ino80 in wild-type, Arp5 KO, and Arp8 KO cells**. **(A)** The chromatin fractions prepared from the indicated cells were immunoblotted with an anti-Ino80 antibody (top panel) and an anti-H3 antibody (lower panel), which was used as an internal control. **(B)** The intensity of the chromatin-bound Ino80 band was normalized with respect to that of H3 band in each case, and the relative amount of chromatin-bound Ino80 in each cell line was normalized with respect to that in the wild-type cells. Student's *t*-test was used to determine the *P*-value. ^*^P < 0.05.

Human Arp8 has been shown to have histone- and DNA-binding activities, which were suggested to contribute to the DSB repair function of the INO80 complex (Kashiwaba et al., 2010; Osakabe et al., 2014; Gerhold et al., 2015). Here, we used Arp8 KO cells to examine whether Arp8 is involved in the binding of INO80 complex to chromatin without inducing DNA damage. When we analyzed the amount of Ino80 in the chromatin fraction prepared from the wild-type, Arp5 KO, and Arp8 KO cells by Western blotting, we observed that the amount of chromatin-bound Ino80 in Arp8 KO cells decreased to around 50% of that in the wild-type cells (Figure 3, Arp8-KO). On the other hand, absence of Arp5 (Arp5 KO cells) did not have any significant effect on the binding of Ino80 to chromatin (Figure 3, Arp5-KO). These results suggested that Arp8, but not Arp5, contributes to the chromatin binding property of the INO80 complex.

### INO80 complex binds to the regulatory sites of *HMOX1*

**Figure 4:**
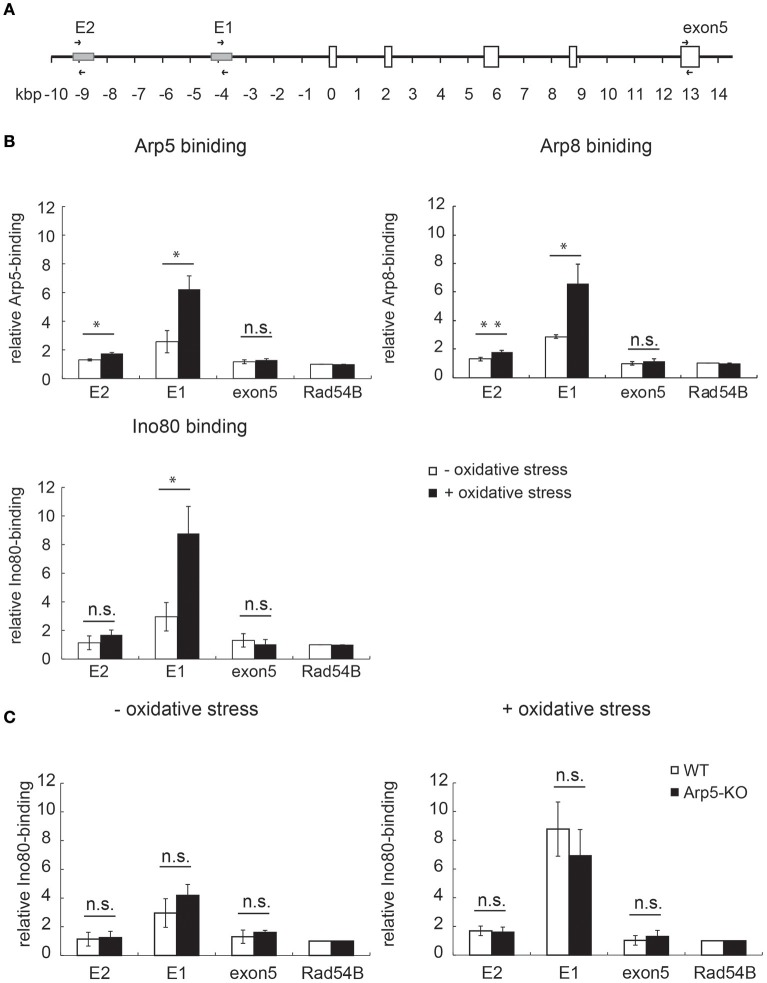
**Analysis of the binding of INO80 complex to the regulatory sites of *HMOX1***. **(A)** Schematic representation of the human *HMOX1* locus. Arrows indicate positions of PCR primer pairs used for the ChIP analysis. **(B)** The quantitative-ChIP assay was performed by using anti-Arp5 (**B**, upper-left panel), anti-Arp8 (**B**, upper-right panel), and anti-Ino80 (**B**, lower-right panel) antibodies. The amount of immunoprecipitated fragment for each site was normalized with respect to the input fraction value, and was shown as relative enrichment to that of the INO80-free Rad54 site (Park et al., 2010). Open and filled bars represent chromatin binding in control (without oxidative stress) and experimental (with oxidative stress) cells, respectively. **(C)** The binding of Ino80 to the regulatory sites in wild-type (WT) and Arp5-KO cells was analyzed by ChIP assay by using an anti-Ino80 antibody in control (without oxidative stress, left panel) and experimental (with oxidative stress, right panel) cells. Open and filled bars represent chromatin-bound Ino80 in WT and Arp5-KO cells, respectively. Data shown are averages from at least three independent experiments (± standard deviation). ^*^P < 0.05 (in **B,C**).

Induction of *HMOX1* expression is under the control of Maf-recognition elements (MARE). Two enhancer regions, one distal (E2) and another proximal (E1), have been identified upstream of the HO-1 coding region (Figure 4A); sequences of both enhancers conform to the sequence of the MARE (Igarashi and Watanabe-Matsui, 2014). To elucidate the role of Arp5 in the activation of *HMOX1* by the INO80 complex, we performed chromatin immunoprecipitation (ChIP) assays using anti-Arp5 (Figure 4B, upper-left panel), anti-Arp8 (Figure 4B, upper-right panel), and anti-Ino80 (Figure 4B, lower-right panel) antibodies. As shown in Figure 4B, in the absence of oxidative stress (open bars), Arp5, Arp8, and Ino80 exhibited slight, but significant binding to the E1 and E2 sites as compared to an INO80-free Rad54B site (Park et al., 2010). The bindings of Arp5 to the E1 and E2 sites in the absence of oxidative stress were confirmed by the comparison of ChIP results with anti-Arp5 and control antibodies (Supplementary Figure S3). After inducing oxidative stress, the bindings of Arp5, Arp8, and Ino80 to the E1 and E2 sites were increased (Figure 4B, filled bars). These results suggested binding of the INO80 complex to the regulatory sites of *HMOX1* in response to oxidative stress.

### Arp5 is not required for the binding of Ino80 to chromatin

To test whether Arp5 is required for the binding of INO80 complex to the regulatory sites of *HMOX1*, ChIP assay was performed using wild-type and Arp5 KO cells and an anti-Ino80 antibody. Interestingly, the binding of Ino80 to the E1 and E2 sites were not impaired in Arp5 KO cells both in the absence and presence of oxidative stress (Figure 4C, left and right panels, respectively). This result suggested that Arp5 is not required for the binding of INO80 complex to the E1 and E2 sites regardless of whether or not the cells were subjected to oxidative stress.

### Arp5 is required for the chromatin remodeling at the regulatory sites of *HMOX1*

**Figure 5:**
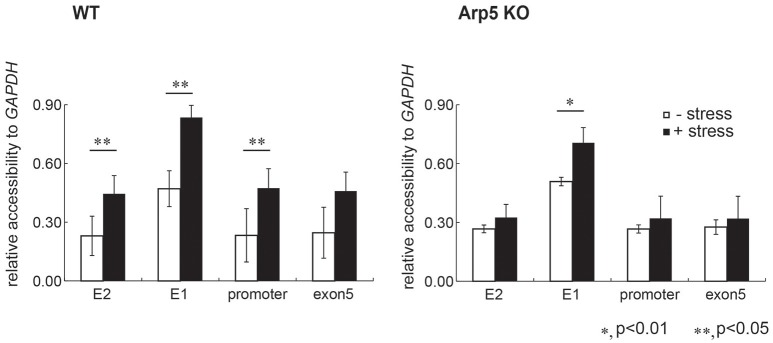
**Comparison of chromatin structure at the regulatory element sites of *HMOX1***. Chromatin is isolated from wild-type (WT) and Arp5 deficient cells, treated with nuclease, genomic DNA is then isolated and chromatin accessibility to nucleases was measured by amplifying the purified DNA using quantitative-PCR. If the chromatin forms a loose structure, the PCR amplification efficiency is low because the genomic DNA is digested by the nuclease. The nuclease accessibility value obtained for each regulatory site of *HMOX1* was normalized with that for the *GAPDH* promoter site, and was shown relative to the nuclease accessibility value obtained for the exon 5 site in control cells (without oxidative stress). Data shown are averages from at least three independent experiments (± standard deviation). ^*^P < 0.05; ^**^P < 0.01.

To test the possibility whether Arp5 is required for the remodeling of chromatin by the INO80 complex, we analyzed the structure of chromatin at the E2 and E1 sites of *HMOX1*. Since open form of chromatin permits accessibility of chromatin DNA to exogenous nuclease digestion, we determined nuclease accessibility of chromatin at the regulatory sites of the wild-type and Arp5 KO cells (Figure 5, left and right panels, respectively) by quantifying the un-cut DNA by qPCR. Under oxidative stress condition, chromatin accessibility to nuclease at the E2 and E1 sites and also at a promoter site adjacent to the E1 site increased similarly in the wild-type cells (Figure 5, left panel). On the other hand, although chromatin accessibility to nuclease at the E2 and the promoter sites did not change upon induction with oxidative stress, the increase in accessibility at the E1 site was partially impaired when Arp5 KO cells were subjected to oxidative stress (Figure 5, right panel). These results suggested that, in response to oxidative stress, the INO80 complex remodels chromatin structure at the regulatory regions of *HMOX1* and that Arp5 is required for this activity of the INO80 complex. At the E1 site, some additional chromatin remodeling complexes might be involved in the change of chromatin structure.

### Arp5 is required for binding of chromatin to the *HMOX1* activator

**Figure 6:**
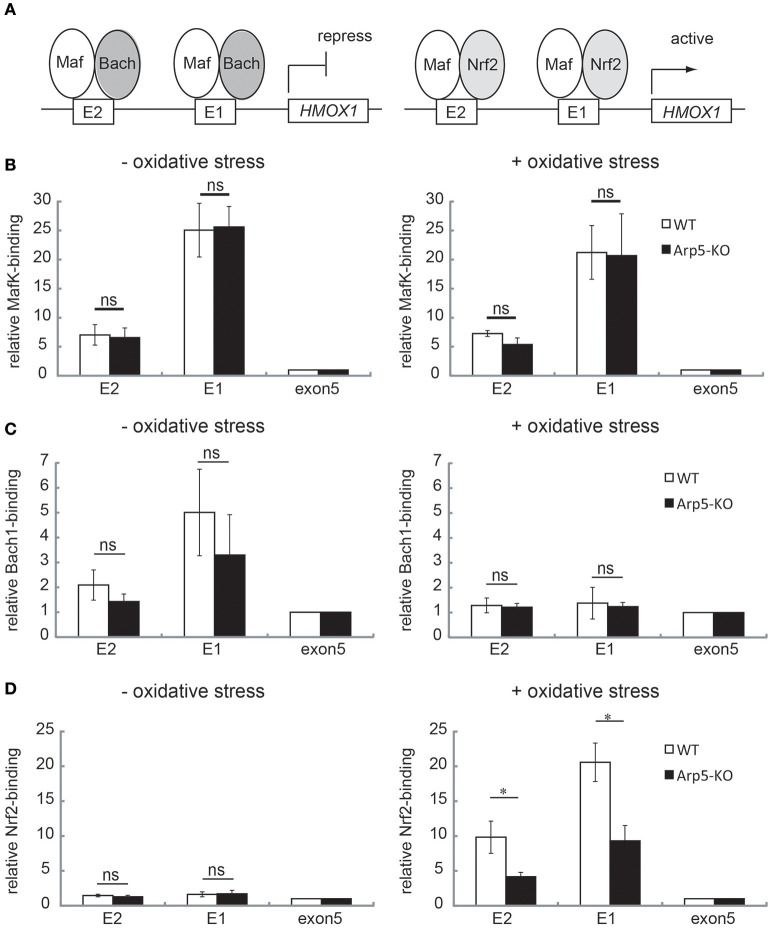
**Analysis of the binding of transcription regulators to the E2 and E1 sites of *HMOX1* in wild-type and Arp5-KO cells**. **(A)** In the absence of any oxidative stress (left panel), the repressor Bach1 forms a heterodimer with MafK, which binds to the Maf recognition element (MARE) at the E2 and E1 sites, and represses *HMOX1* transcription. In the presence of oxidative stress (right panel), Bach1 is released, and the activator Nrf2 forms a heterodimer with MafK and binds to the MARE elements at E2 and E1. To analyze the binding of these transcriptional regulators, quantitative-ChIP assay was performed using antibodies against MafK **(B)**, Bach1 **(C)**, and Nrf2 **(D)** in wild-type (open bar) and Arp5-KO (filled bar) cells. The amount of immunoprecipitated fragment was normalized with respect to the input fraction value. Data shown are binding relative to that of exon 5. Averages from at least three independent experiments (± standard deviation) are shown. *P*-value (Student's *t*-test) for the difference between WT and Arp5 KO cells is indicated. ^*^P < 0.05.

In the transcriptional regulation of *HMOX1*, a small Maf oncoprotein, MafK, directly binds to MARE sequences at the E1 and E2 sites of *HMOX1*, and regulates both repression and activation of *HMOX1* expression depending on its dimeric partner (Igarashi and Watanabe-Matsui, 2014). In the absence of oxidative stress, the repressor Bach1 associates with the E1 and E2 sites by forming a heterodimer with MafK and represses *HMOX1*. On the other hand, under oxidative stress, Bach1 is released from the chromatin when the NF-E2-related factor 1 (Nrf2) associates with the E1 and E2 sites by forming a heterodimer with MafK, which remains on the chromatin, and activates *HMOX1* (Figure 6A). To examine the involvement of Arp5 in the association and/or dissociation processes of these transcription factors in the induced activation of *HMOX1* by oxidative stress, ChIP assays were carried out using the wild-type and Arp5 KO cells and specific antibodies against these transcription factors. Both in wild-type and Arp5 KO cells, consistently similar level of MafK binding was observed at the E1 and E2 sites under both control (no oxidative stress) and oxidative stress conditions (Figure 6B).

In wild-type cells, as was reported previously (Igarashi and Watanabe-Matsui, 2014), the association of Bach1 to the E1 and E2 sites was observed under no oxidative stress condition (Figure 6C, left panel), but not under oxidative stress condition (Figure 6C, right panel). In Arp5-KO cells, the association and dissociation of Bach1 were not disturbed (Figure 6C, Arp5-KO).

The binding of Nrf2 to E2 and E1 sites increased in response to oxidative stress in wild-type cells (Figure 6D, WT), as was reported previously (Igarashi and Watanabe-Matsui, 2014). In contrast, the binding of Nrf2 to E2 and E1 sites in response to oxidative stress was significantly impaired in Arp5-KO cells (Figure 6D, Arp5-KO). However, the total amount of Nrf2 and Mafk proteins in the Arp5 KO cells was similar to that in the wild-type cells (Supplementary Figure S4). Taken together with the observations that Arp5 binds to the E1 and E2 sites even in the absence of oxidative stress and that its binding to these sites was increased in response to the stress, these results tend to suggest that Arp5-dependent remodeling of chromatin by the INO80 complex is required for the binding of Nrf2 to the regulatory sites of *HMOX1*.

## Discussion

### Roles of actin family proteins in the INO80 complex

By using Arp5 KO and Arp8 KO cells, we have been able to successfully demonstrate here that Arp5 and Arp8 have roles in the INO80 chromatin remodeling complex, contributing to the induction of *HMOX1* by oxidative stress. We previously reported that the conditional KO of Arp8 leads to cell lethality (Osakabe et al., 2014). In the present study, we demonstrated that KO of Arp5 causes cells to grow slow, but does not cause cell lethality (Supplementary Figure S2). These observed differences in phenotypes of Arp5 KO and Arp8 KO cells support the contention that the actin family proteins Arp5 and Arp8 have distinctive roles in the INO80 complex.

Although the structural details of the human INO80 complex are not known yet, the fact that the components of the yeast and human INO80 complexes are evolutionarily conserved implies a high degree of structural similarity between these complexes (Gerhold and Gasser, 2014). Arp8, but not Arp5, is included in the Arp8 module together with actin and Arp4 (Tosi et al., 2013). The Arp8 module directly associates with the helicase-SANT-associated (HSA) domain of the enzymatic Ino80 ATPase scaffold. Arp8 has both histone- and DNA-binding activities (Gerhold et al., 2012; Osakabe et al., 2014), and Arp4 also has histone binding activity (Harata et al., 1999; Nishimoto et al., 2012). Although actin itself has neither histone- nor DNA-binding activity, actin in the budding yeast INO80 complex is required for the binding of the INO80 complex to extranucleosomal DNA (Bartholomew, 2013; Kapoor et al., 2013). Consistent with this observation, a subcomplex comprising of the actin family molecules belonging to the Arp8 subdomain (actin, Arp4, and Arp8) and the HSA domain fragment of Ino80 exhibited significantly more affinity for DNA binding than expected from the individual subunits (Gerhold et al., 2012). These observations suggested that, despite displaying different characteristics, actin family proteins in the Arp8 module (i.e., actin, Arp4, and Arp8) function in a cooperative manner so that the INO80 complex could bind to chromatin.

Arp5 is also shown to be required for the function of the INO80 complex in budding yeast (Yao et al., 2015, 2016). Arp5 is included in the Arp5 module, which indirectly associate with the enzymatic Ino80 ATPaes scaffold through the Rvb module (Tosi et al., 2013). In addition, Arp5 by itself, unlike Arp8, is not able to bind to either DNA or histone. The distinctive functions of Arp5 and Arp8 in the INO80 complex may rise from their topological distribution on the INO80 complex and differences in their biochemical characters.

### A model depicting the function of Arp5 and Arp8 in *HMOX1* expression

**Figure 7:**
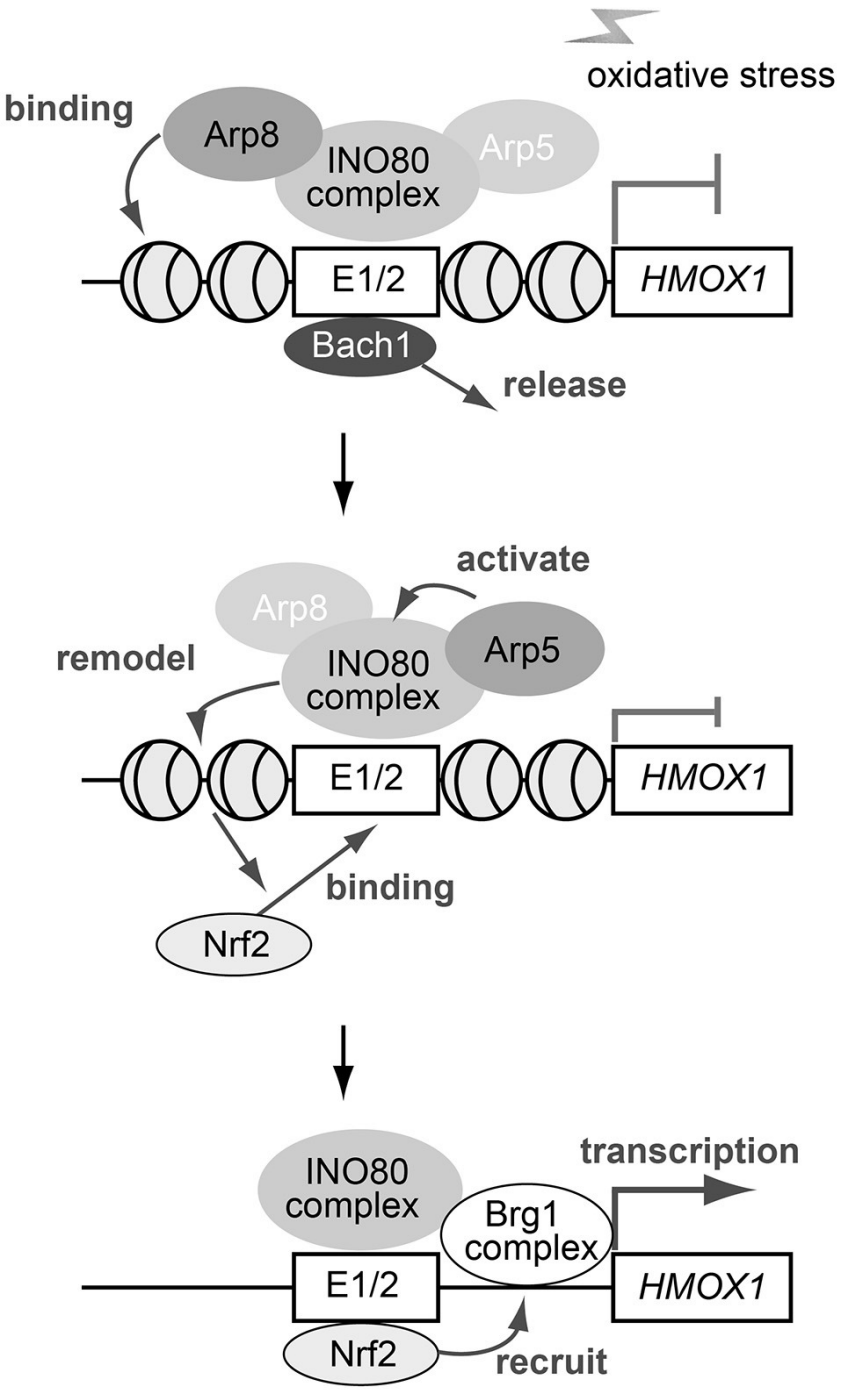
**A schematic model depicting the roles of Arp5 and Arp8 in the oxidative stress-induced expression of *HMOX1***. See text for details.

Based on our observations, together with the results described in previous reports (Zhang et al., 2006; Maruyama et al., 2013; Igarashi and Watanabe-Matsui, 2014), we propose a model depicting the roles Arp5 and Arp8 might play in the oxidative stress-induced activation of *HMOX1* (Figure 7). In the proposed model, the INO80 complex initially binds to the regulatory sites of *HMOX1* with the help of Arp8, but not that of Arp5, in response to oxidative stress. The repressor Bach1 is released from the regulatory sites independently of the activity of the INO80 complex (top). Next, the chromatin remodeling activity of the INO80 complex is induced with the help of Arp5. The remodeled chromatin structure induces the biding of the activator Nrf2 to the regulatory sites (middle). Finally, another chromatin remodeling complex BRG1 has been reported to be recruited to these sites in an Nrf2-dependent manner (Zhang et al., 2006; Maruyama et al., 2013). The BRG1 complex is shown to alter the B-DNA structure at the *HMOX1* promoter to Z-DNA structure (Maruyama et al., 2013; Zhang et al., 2006). Z-DNA formation reduces nucleosome occupancy and induces RNA polymerase II recruitment to the *HMOX1* promoter, and thereby activates the transcription of *HMOX1* (bottom).

### Involvement of nuclear actin family proteins in the maintenance of genome integrity

The oxidative stress causes injury to genome DNA, and this leads to carcinogenesis, Alzheimer, aging, and various other diseases (Ryter et al., 2006; Klaunig et al., 2010; Coppedè and Migliore, 2015; Foppoli et al., 2015; Loboda et al., 2016). Oxidative stress inducible genes help in protecting the genome from the stress. For example, HO-1 has a strong reducing capacity and removes substances responsible for ROS formation. Therefore, the INO80 complex appears to play a role in the maintenance of genome integrity by activating oxidative stress inducible genes including *HMOX1*. It is also known that the INO80 complex helps maintaining the genome stability via its roles in DNA replication and DSB repair (Kawashima et al., 2007; Shimada et al., 2008; Kashiwaba et al., 2010; Gerhold et al., 2015). Based on the known broad functions of the INO80 complex in stress response and DNA metabolism, it could be conferred that Arp5 and Arp8, possibly together with actin and Arp4, may have indispensable roles in the maintenance of genome integrity.

## Author contributions

YT, HM, YA, YK, YO, HN, and KS contributed to the acquisition, analysis, and interpretation of data. KI and MH made substantial contribution to the conception and design of the work. All authors participated in drafting the manuscript, approval for publication, and agree to be accountable for all aspects of this work.

### Conflict of interest statement

The authors declare that the research was conducted in the absence of any commercial or financial relationships that could be construed as a potential conflict of interest.
