# Supplementary figures and images for: Actin Family Proteins in the Human INO80 Chromatin Remodeling Complex Exhibit Functional Roles in the Induction of Heme Oxygenase-1 with Hemin
Source: Front Genet. 2017 Feb 21;8:17. doi: 10.3389/fgene.2017.00017 (PMC5318382; doi:10.3389/fgene.2017.00017)

ARP5 (Chr 20: 23737 bp)

$\Delta$  259-392 aa

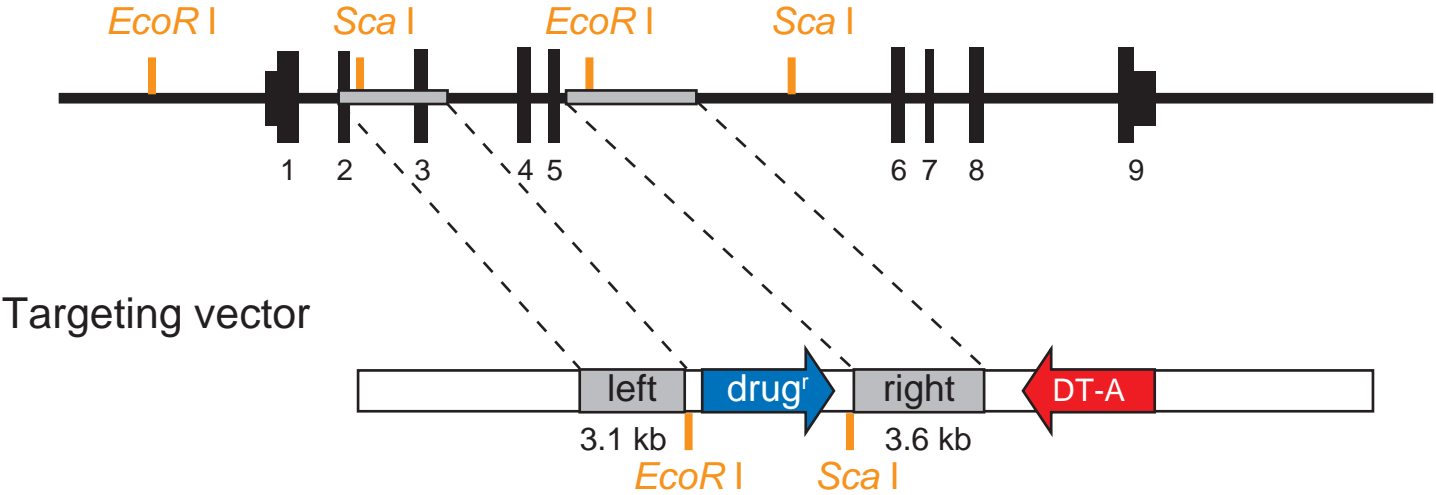

Supplementary Fig. S1

**A**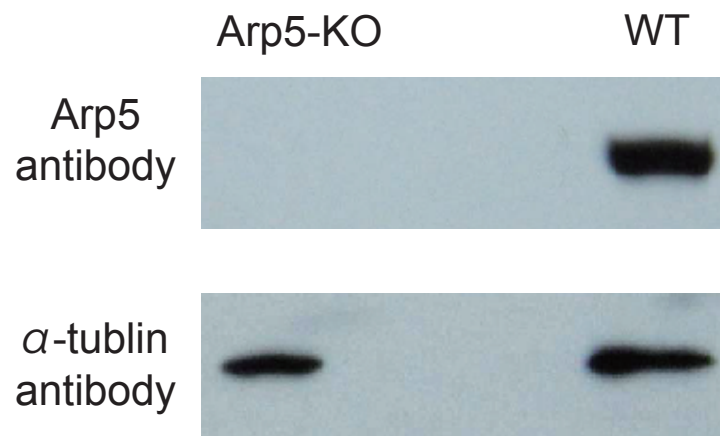**B**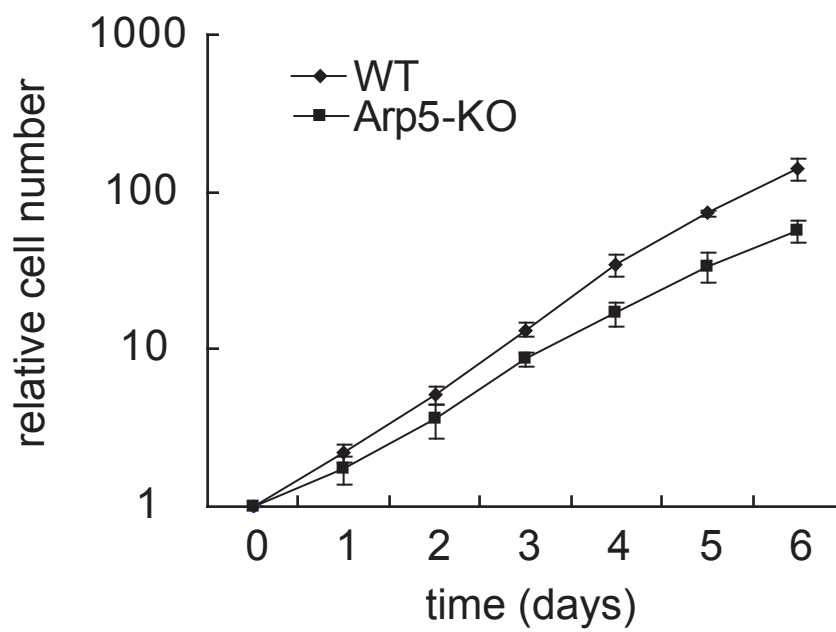

Suppl. Fig. S2

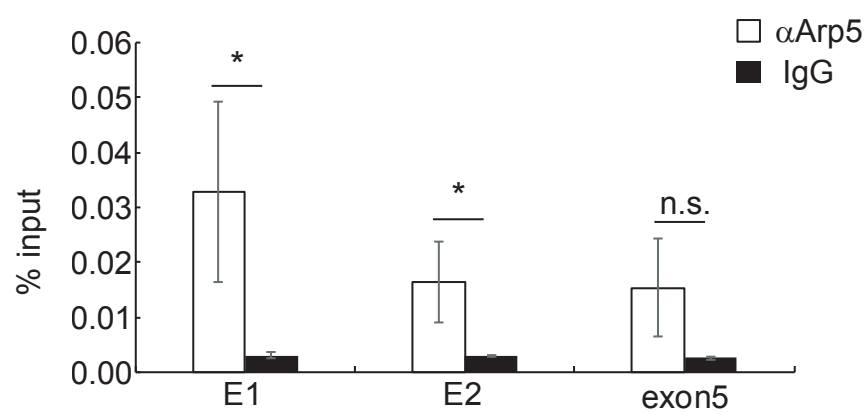

**Supplementary Fig. S3**

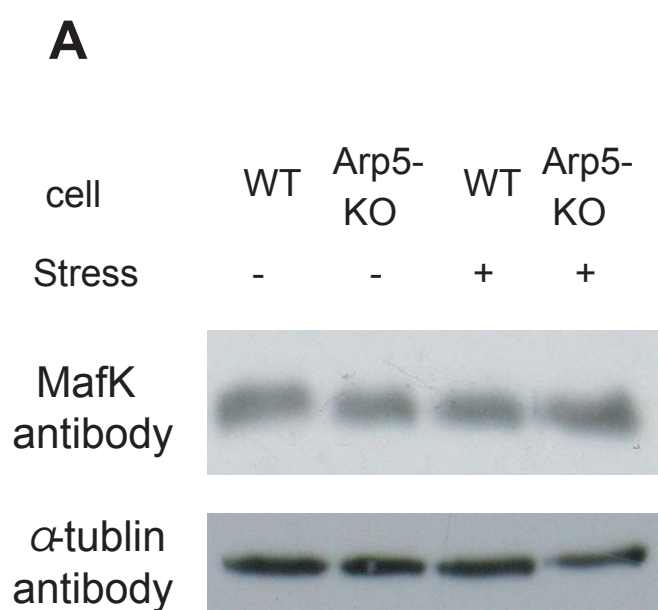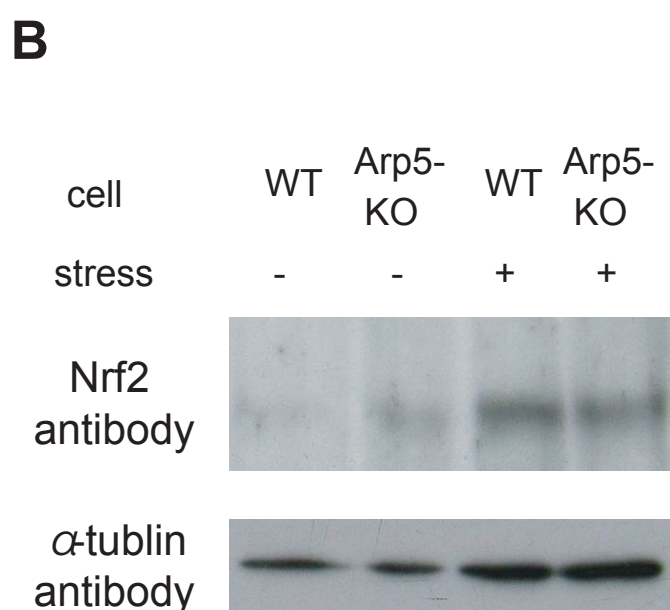

Supplementary Fig. S4

Supplement: Supplementary file 11 [file Image1.PDF]
