# Supplementary material for: Actin Family Proteins in the Human INO80 Chromatin Remodeling Complex Exhibit Functional Roles in the Induction of Heme Oxygenase-1 with Hemin
Source: Front Genet. 2017 Feb 21;8:17. doi: 10.3389/fgene.2017.00017 (PMC5318382; doi:10.3389/fgene.2017.00017)
Supplement: Supplementary file 12 [file DataSheet1.DOCX]

**SUPPLEMENTARY INFORMATION**

**Supplementary Fig. S1.** Establishment of Arp5 gene knockout cells. Restriction maps of the Arp5 knockout targeting vector construct and gene locus following the targeted integration of the Arp5 knockout construct. Black boxes (upper) indicate the positions of exons. The targeting vector construct is expected to disrupt two exons.

**Supplementary Fig. S2**. Western blot analysis and growth curves of Arp5-knockout cells. (A) Whole-cell extracts prepared from the wild-type (WT) and Arp5-KO cells were analyzed by Western blot using either an anti-Arp5 or an anti-α-tubulin antibody. (B) Representative growth curves for the wild-type (WT, 0- to 6-day) and Arp5-KO cells (0- to 6-day). Y-axis indicates cell number in log-scale. The number of living cells was counted by trypan blue staining and was shown in the plot as relative cell number.

**Supplementary Fig. S3**. The quantitative-ChIP assay was performed by using an anti-Arp5 antibody (open bar) or a control IgG (filled bar). Data shown are averages from at least three independent experiments (± standard deviation). *, P<0.05.

**Supplementary Fig. S4.** Expression levels of *HMOX1* transcription factors. Whole-cell extracts were prepared from the wild-type (WT) and Arp5 KO cells cultured in the absence (-) or presence (+) of 20 µM heme for 24 hrs, and expression levels of transcription factors MafK and Nrf2 were analyzed by Western blotting with (A) an anti-MafK or (B) an anti-Nrf2 antibody. An anti-α-tubulin antibody was used as a control.
